# Supplementary material for: TOM40 Inhibits Ovarian Cancer Cell Growth by Modulating Mitochondrial Function Including Intracellular ATP and ROS Levels
Source: Cancers (Basel). 2020 May 22;12(5):1329. doi: 10.3390/cancers12051329 (PMC7281007; doi:10.3390/cancers12051329)
Supplement: Supplementary file 1 [file cancers-12-01329-s001.pdf]

Supplementary Materials

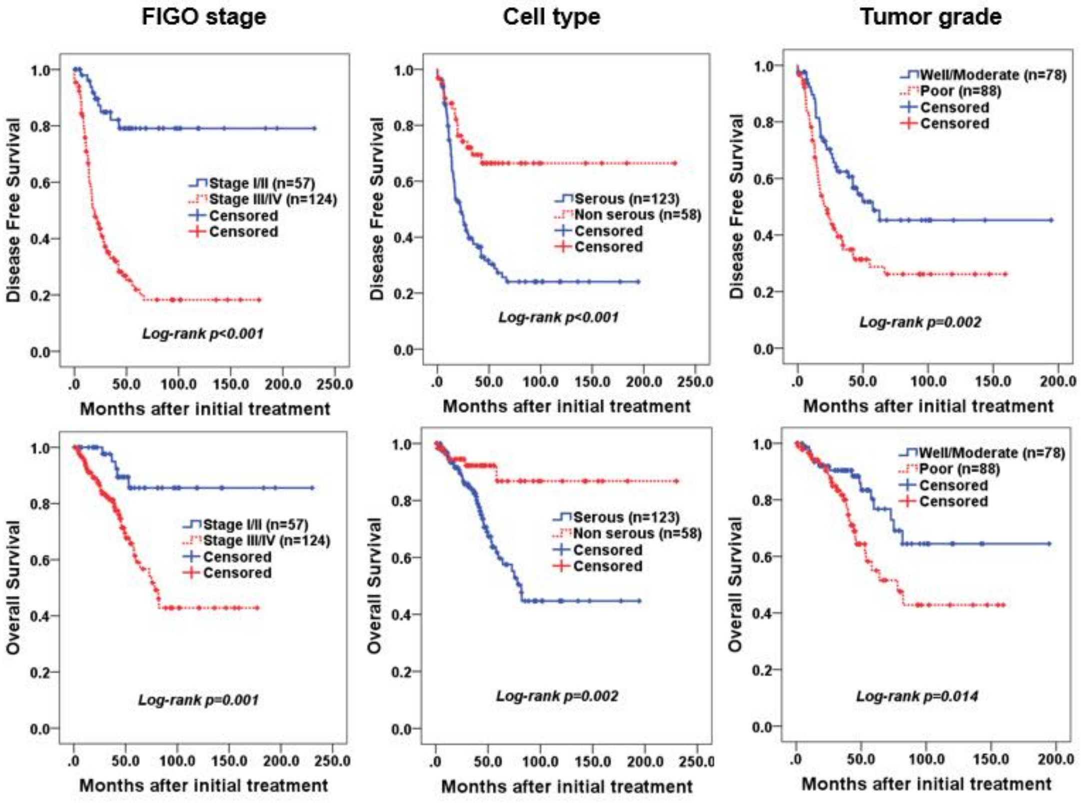

Figure S1. Disease-free survival and overall survival curves for patients with epithelial ovarian cancer (EOC) according to FIGO stage, cell type, and tumor grade. FIGO, The International Federation of Gynecology and Obstetrics.

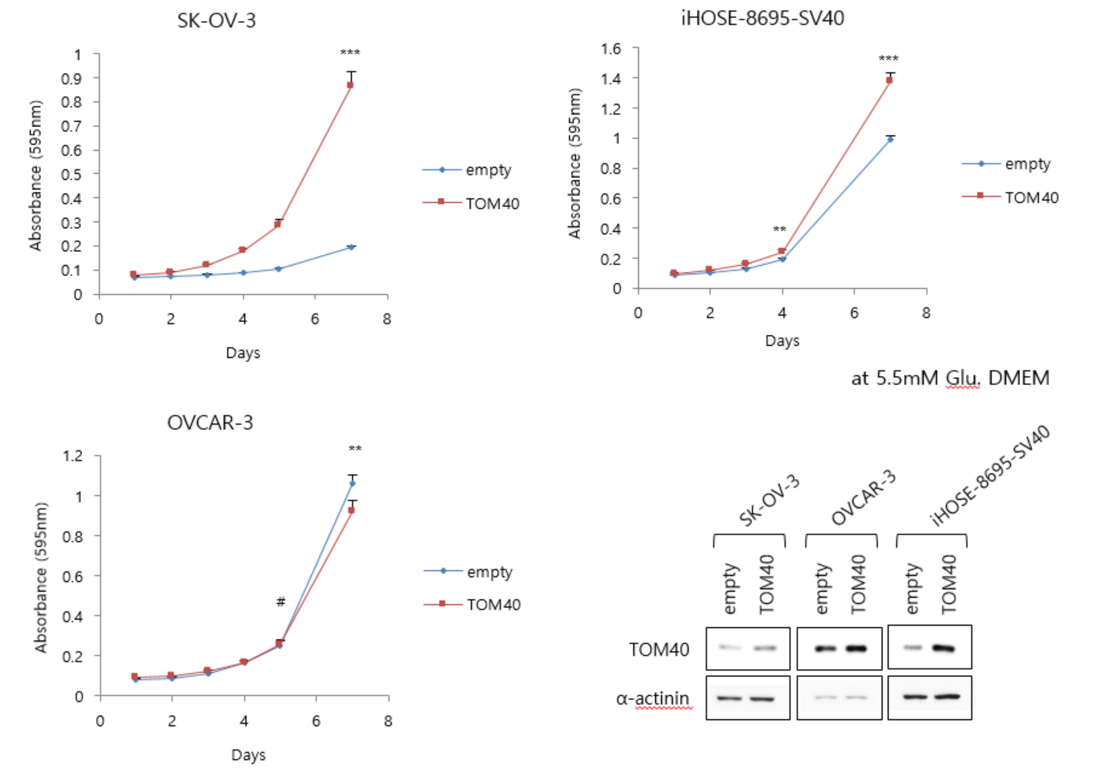

Figure S2. The growth rate for TOM40 stably overexpressed cells. EOC cells (SK-OV-3, OVCAR-3, and iHOSE-8695-SV40) that stably express pCDH-empty or pCDH-TOM40 were established by lentivirus and selected by 3 μg/ml puromycin for 15 days. The comparative growth rate was measured daily from day 1 through 7, post-seeding, with a crystal violet assay for pCDH-empty vs. pCDH-TOM40 EOC cells. Data are expressed as the mean ± S.D., n = 4;). $^{\#}$ $p > 0.05$, ** $p < 0.01$, *** $p < 0.001$. Overexpression of TOM40 was examined by western blot analysis using anti-TOM40 antibody (right-bottom panel).

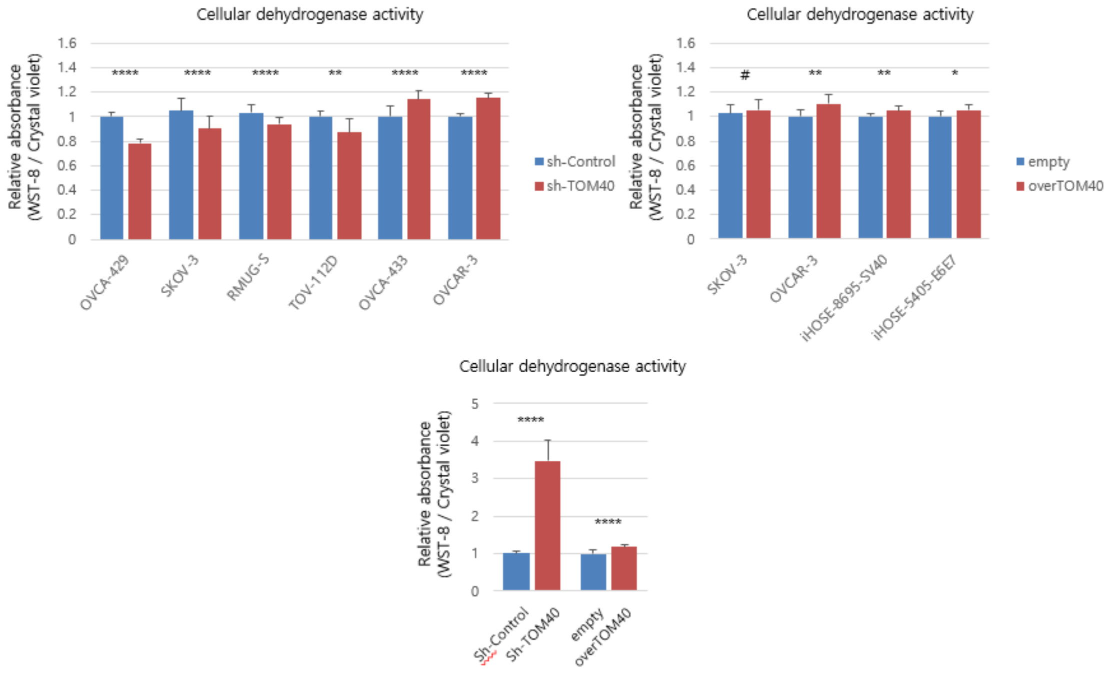

Figure S3. The measurement of intracellular dehydrogenase activity according to TOM40 expression. The stably expressed EOC cells and HEK293T cells were seeded 1×10^{4} cells per well in 96 well plate.

The cells were stained WST-8 solution for 2 h and were measured at 450 nm and 600 nm using ELISA reader. Then, the cells were stained crystal violet solution for the relative cell counting and were detected the absorbance at 595 nm by ELISA reader. The relative cellular dehydrogenase activity were quantified as a ratio of the means of WST-8 absorbance at 450 nm / crystal violet absorbance at 550 nm. The bar graph expressed as the mean of relative absorbance ± S.D., n = 12. $^{\#}$ $p > 0.05$; * $p < 0.05$; ** $p < 0.01$; **** $p < 0.0001$.

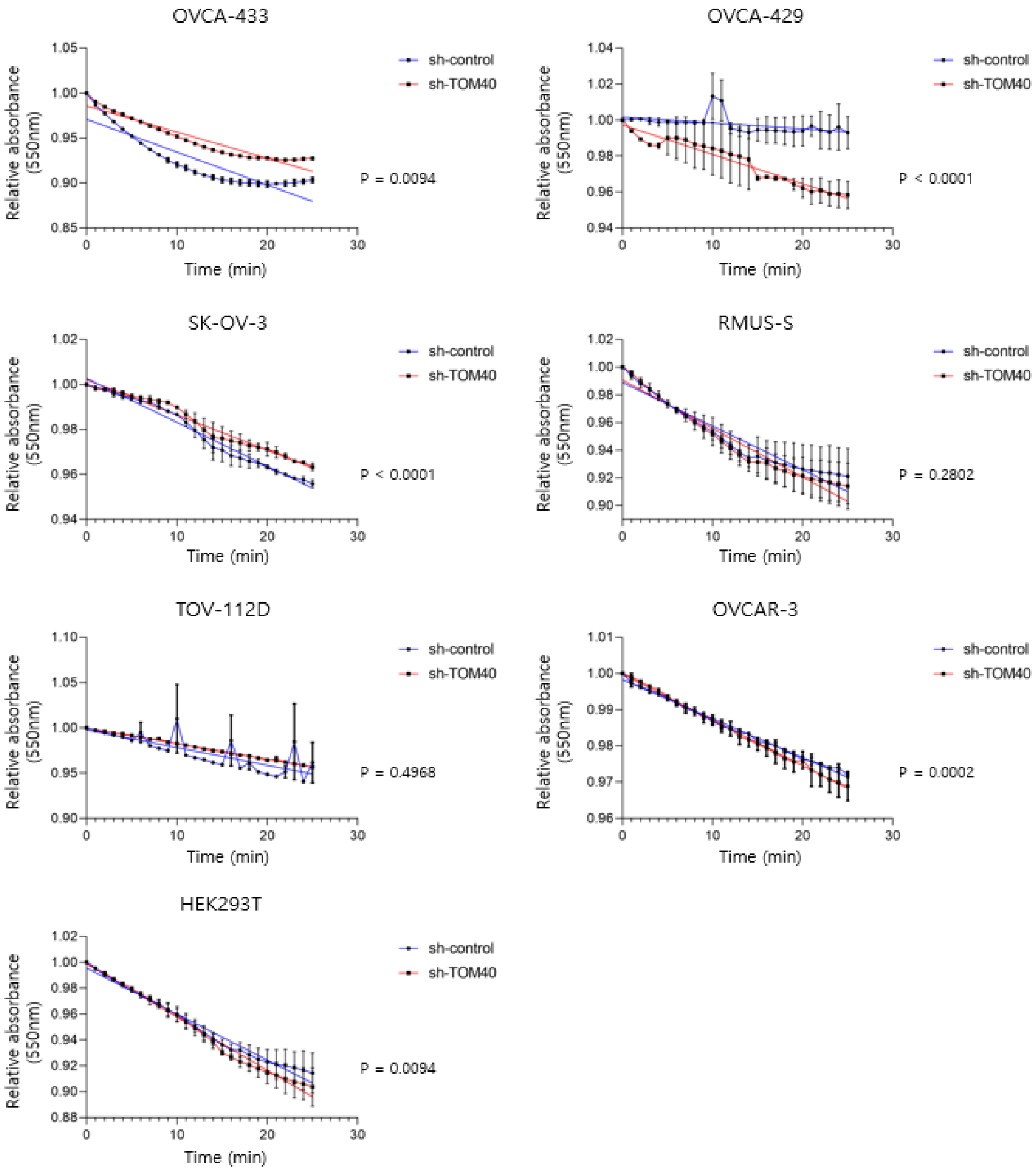

Figure S4. Cytochrome oxidase activity is not correlated with TOM40 expression in various cell lines. EOC and HEK293T cells that stably express sh-Control and sh-TOM40 were harvested over 70% confluence, and whole cell lysates were extracted using protein lysis buffer. The cytochrome oxidase activity inside the cell lysate was measured by the cytochrome oxidase activity assay kit. The kinetic enzyme activity was measured at 550 nm using an ELISA reader (The interval time is 1 min, and the temperature for the enzyme reaction is at 25 °C). Data are expressed as the mean of fold change ± S.E., $n = 2$. The p value means the significant differences between the two slopes.

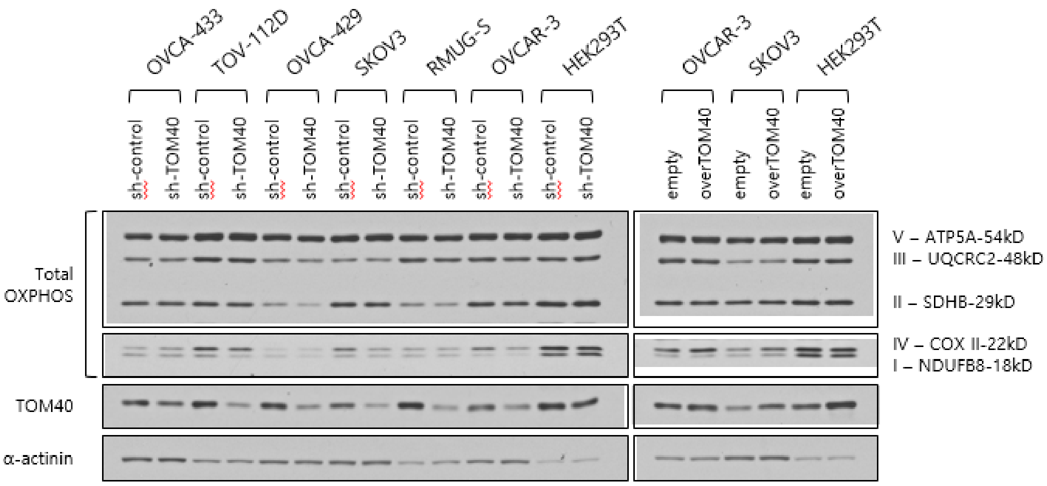

Figure S5. Five subunits of total OXPHOS are slightly or not changed according to TOM40 expression. The stably expressed EOC cells and HEK293T cells were harvested over 70% confluence, and whole cell lysates were extracted using protein lysis buffer. The expression levels of indicated proteins were detected by the western blot analysis.

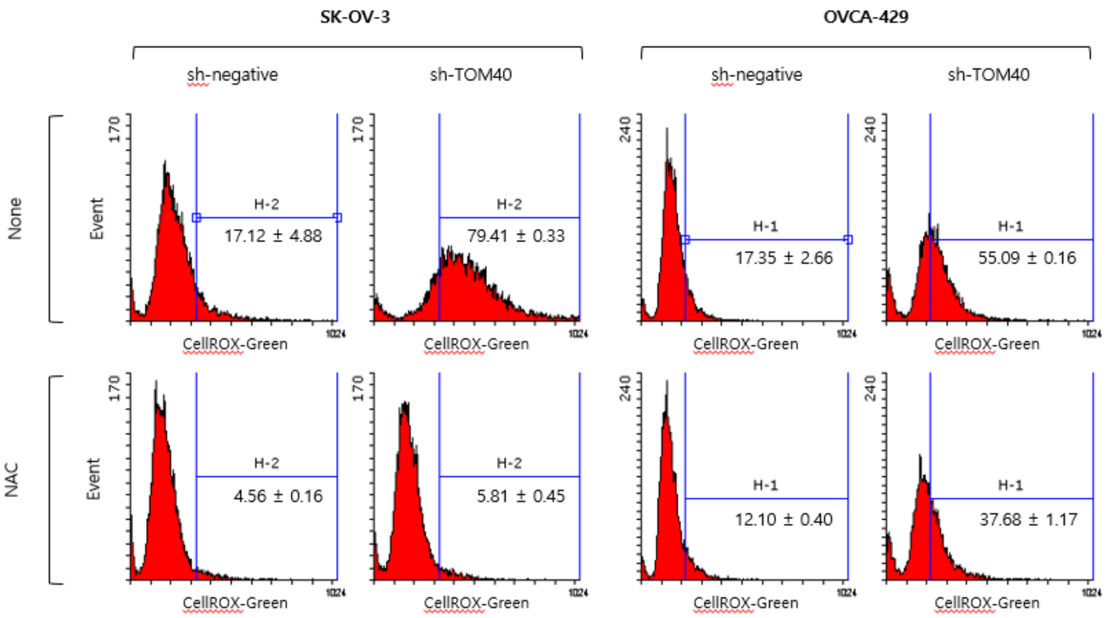

Figure S6. The intracellular reactive oxygen species increase in TOM40 knock-down cells comparing with sh-control expressed cells. Representative images of FACS analysis of CellROX-Green staining to measure intracellular ROS levels. Results represent as the means ± S.E. n = 2. NAC is N-acetyl-L-cystein as the antioxidant.

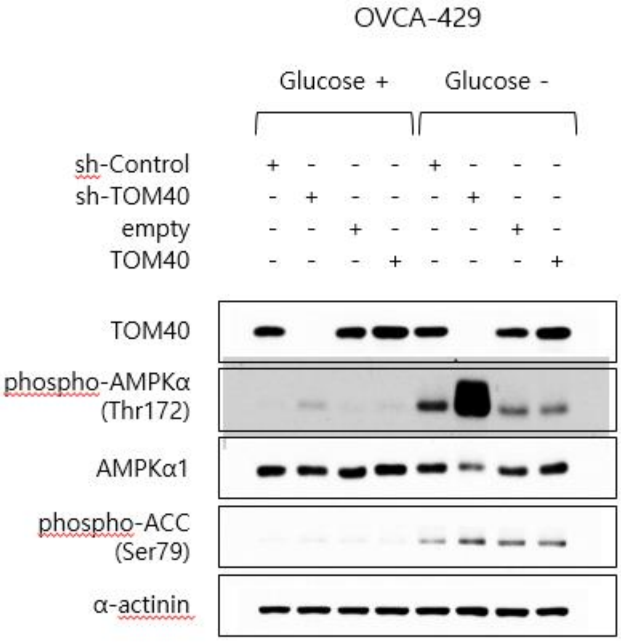

Figure S7. TOM40 expression negatively correlates with AMPK activity in OVCA-429 cells. OVCA-429 cells that stably express sh-Control, sh-TOM40, empty vector, or TOM40 were cultured in DMEM with 25 mM glucose or 0 mM glucose for 24 h before cell lysates were harvested. The expression levels of indicated proteins were examined by western blot analysis.

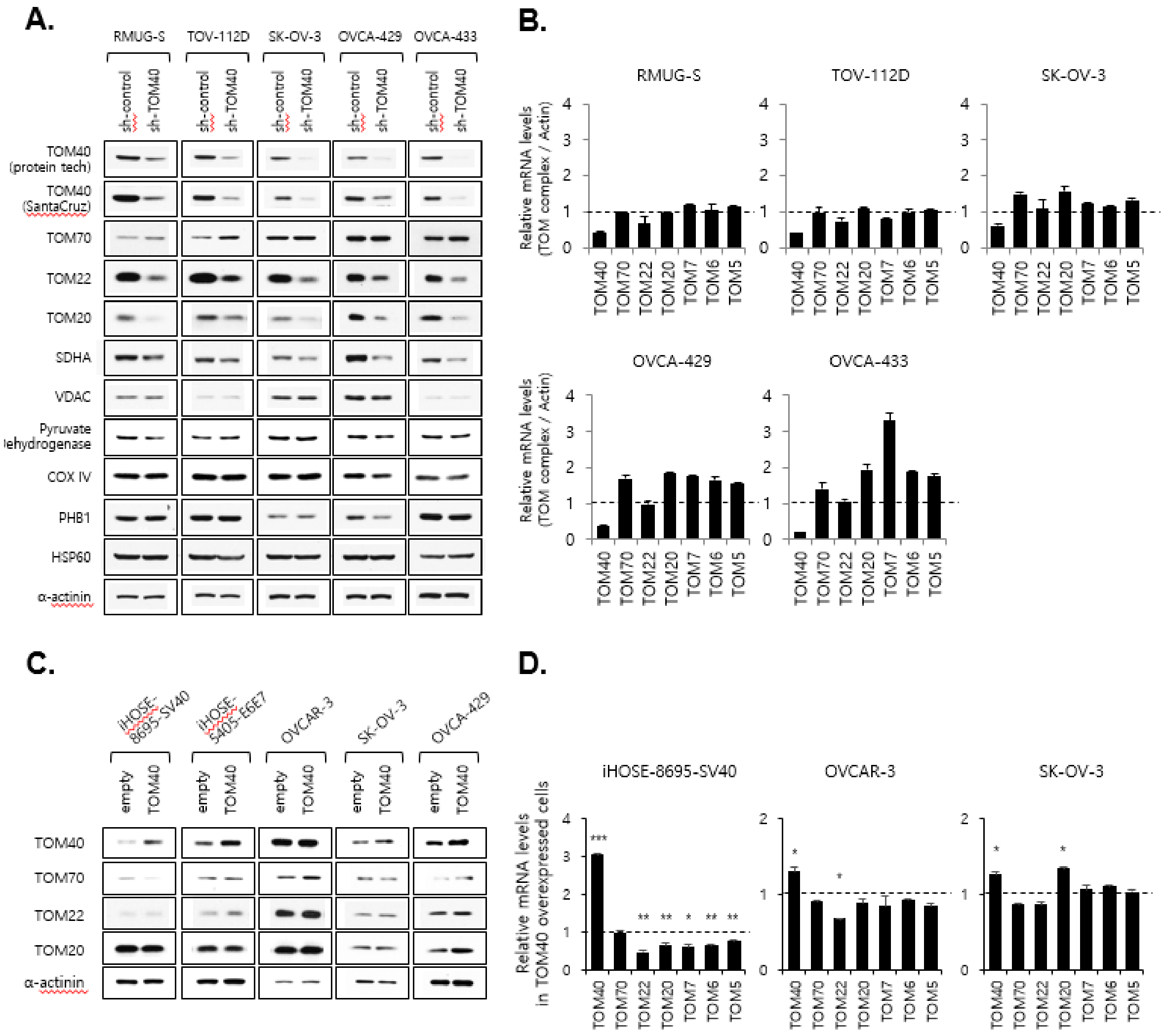

Figure S8. The protein levels of TOM complex subunits TOM22 and TOM20 decrease with TOM40 knockdown in epithelial ovarian cancer (EOC) cell lines. (**A**) EOC cells that stably express sh-TOM40 or sh-Control were harvested at 70% confluence, and whole cell lysates were extracted using protein lysis buffer. The expression levels of indicated proteins were measured by western blot analysis. (**B**) The mRNA levels of TOM complex subunits were measured by real-time polymerase chain reaction (PCR) in indicated EOC cell lines that stably express sh-TOM40 or sh-Control. Fold induction is expressed as the ratio of TOM subunit mRNA/actin mRNA, and compares sh-TOM40-expressing cells to sh-Control-expressing cells. Data represent the means ± S.E., $n = 3$. (**C**) The expression levels of TOM40, TOM70, TOM22 and TOM20 by western blot analysis in various EOC cell lines and immortalized HOSE cells (iHOSE) that stably express empty vector or TOM40. Band intensities of TOM40 were quantified as a ratio to α-actinin using Image J 1.48v software (fold change below TOM40 protein bands). (**D**) The mRNA levels of TOM complex subunits were measured by real-time PCR in EOC cell lines and iHOSE cells that stably express empty vector or TOM40. Fold induction is expressed as a ratio to TOM subunit mRNA/actin mRNA and compares empty vector-expressing cells to TOM40-expressing cells. Data represent the means ± S.E., $n = 3$; * $p < 0.05$, ** $p < 0.01$, *** $p < 0.001$.

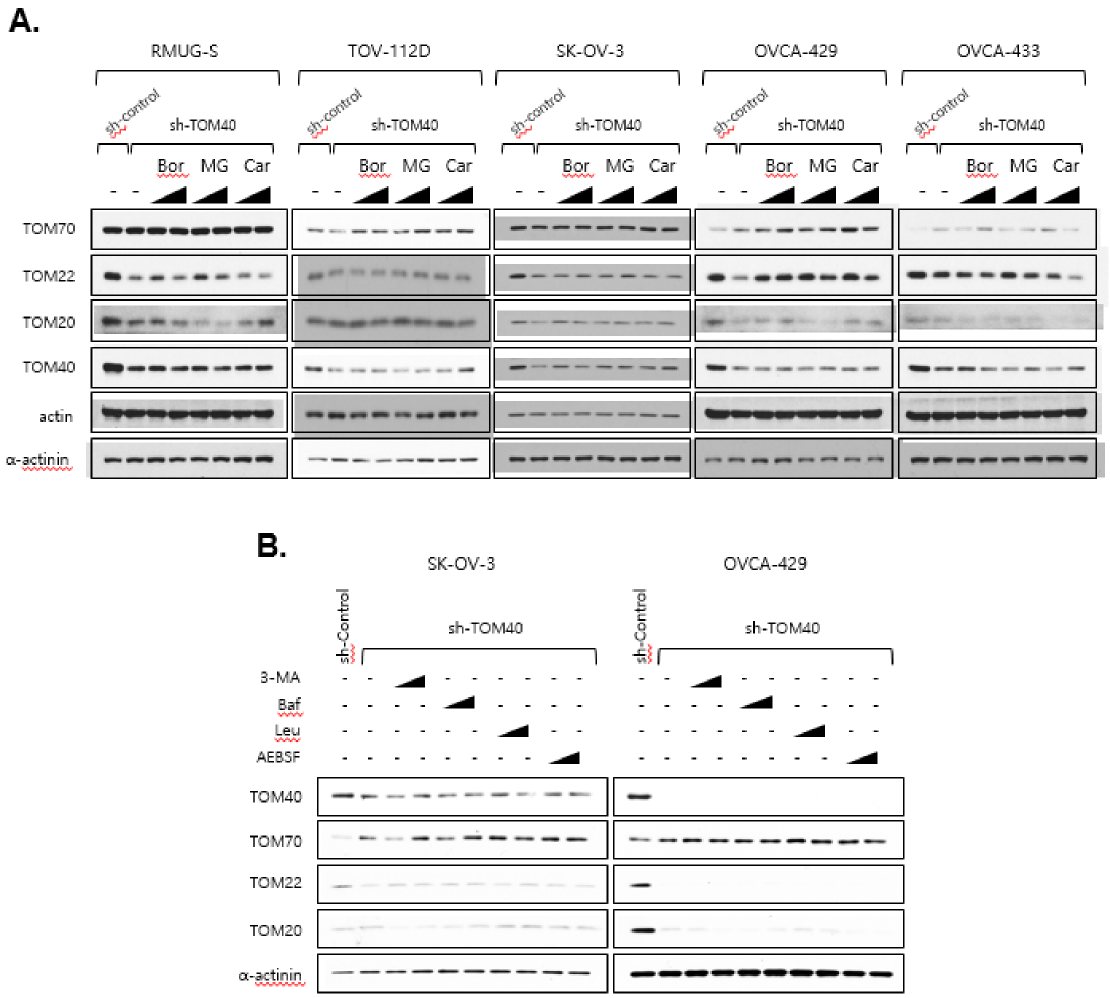

Figure S9. The molecular mechanism by which TOM40 regulates the expression of other TOM complex subunits has not been determined. (**A**,**B**) Western blot analyses of the expression of TOM complex subunit proteins by epithelial ovarian cancer (EOC) cell lines that stably express sh-control or sh-TOM40. (**A**) Cells were treated with the following proteasome inhibitors for 6 h. Bortezomib (Bor), 100 nM and 250 nM; MG132 (MG), 1 μM and 2.5 μM; Carfilzomib (Car), 100 nM and 250 nM. (**B**) Cells were treated with the following lysosome inhibitors and protease inhibitors for 24 h. 3-methyladenine (3-MA), 0.5 mM and 2.5 mM; bafilomycin (Baf), 10 nM and 50 nM; leupeptin (Leu), 25 μM and 100 μM; 4-(2-aminoethyl) benzenesulfonyl fluoride hydrochloride (AEBSF), 50 μM and 250 μM.

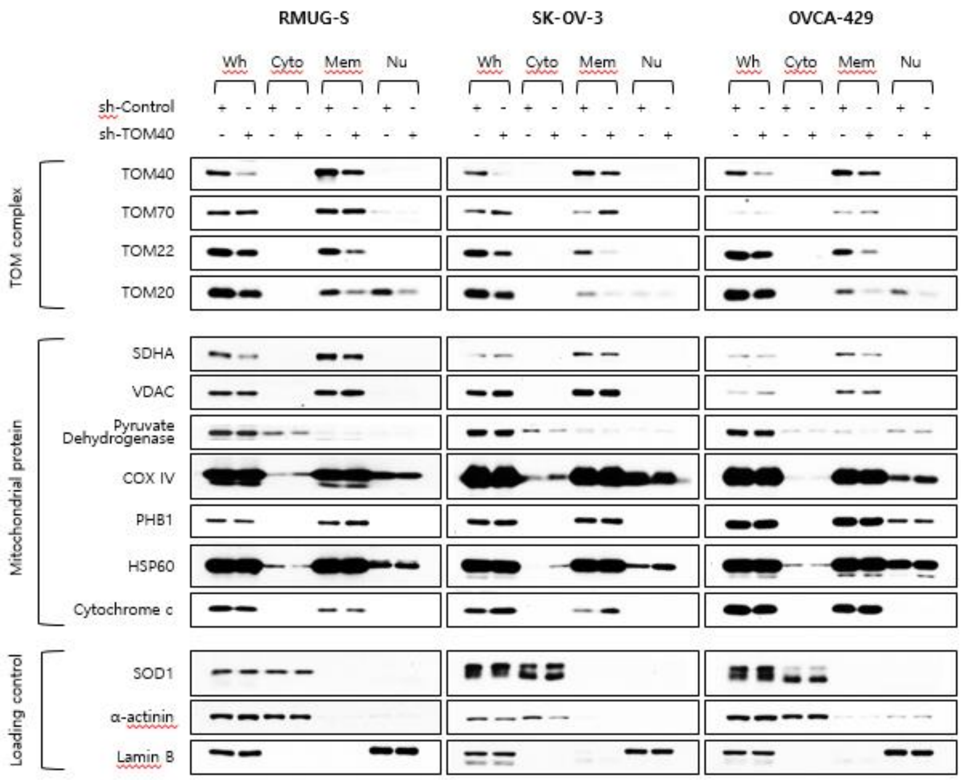

Figure S10. TOM40 knockdown does not promote the translocation of several mitochondrial proteins to the mitochondria. Expression and localization of TOM complex subunits and mitochondrial proteins by western blot analysis. EOC cell lines that stably express sh-Control or sh-TOM40 were harvested at 70% confluency, and divided into subcellular fractions. Whole cell lysates (Wh), cytoplasmic fraction (Cyto), organellular/membrane (Mem), nuclear/cytoskeletal fraction (Nu).

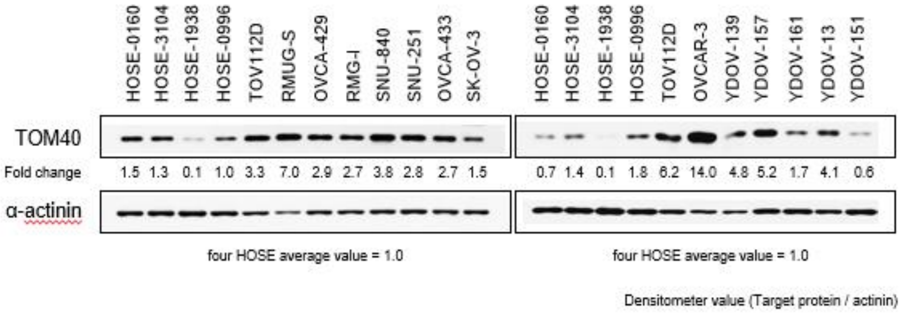

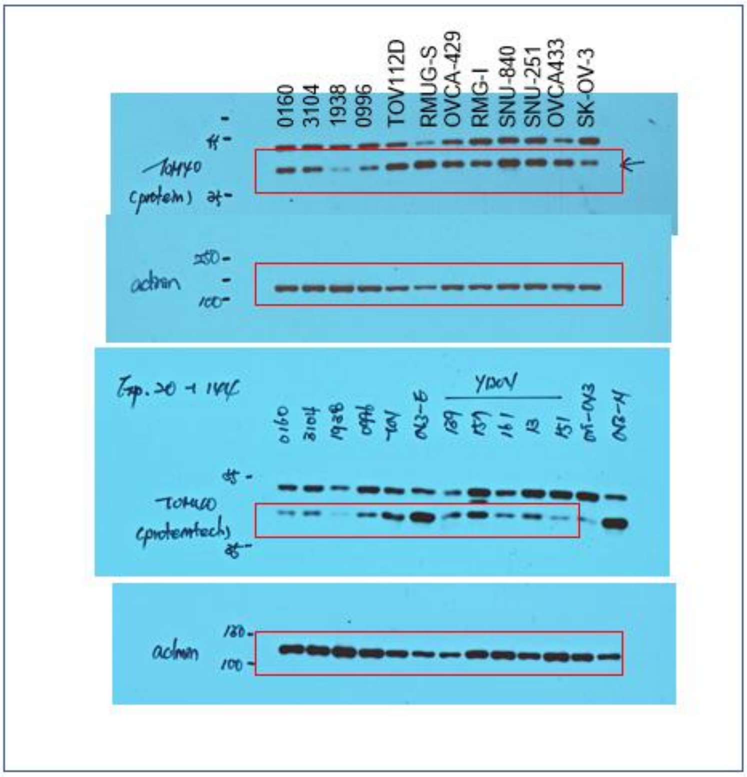

Figure S11. Original Western blots of figure 1B.

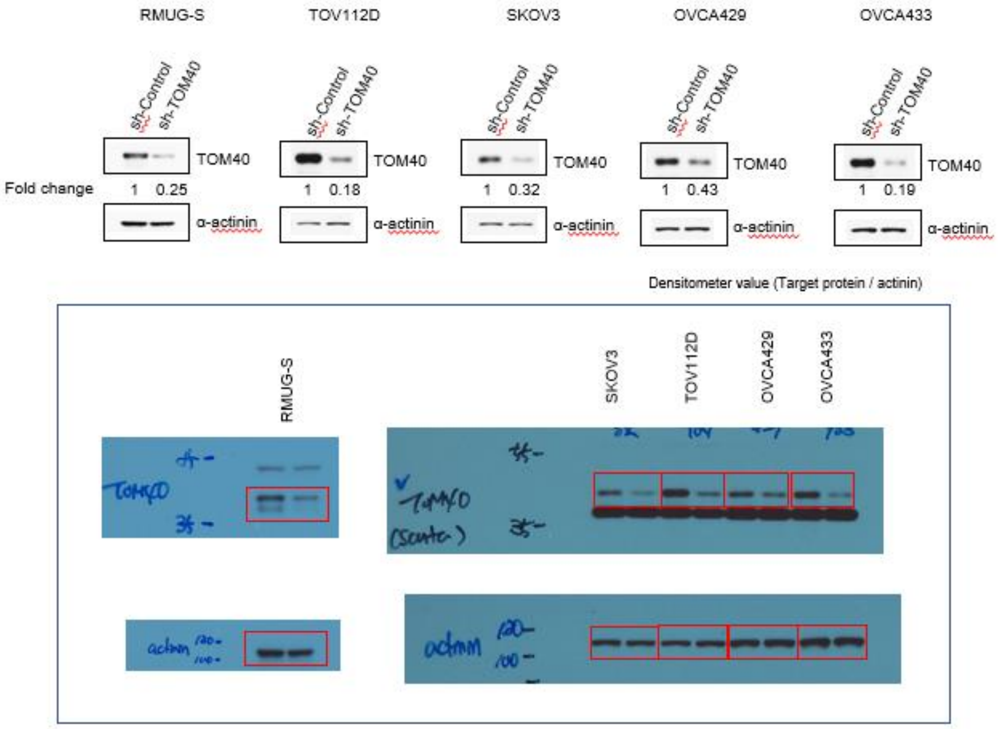

Figure S12. Original Western blots of figure 2A.

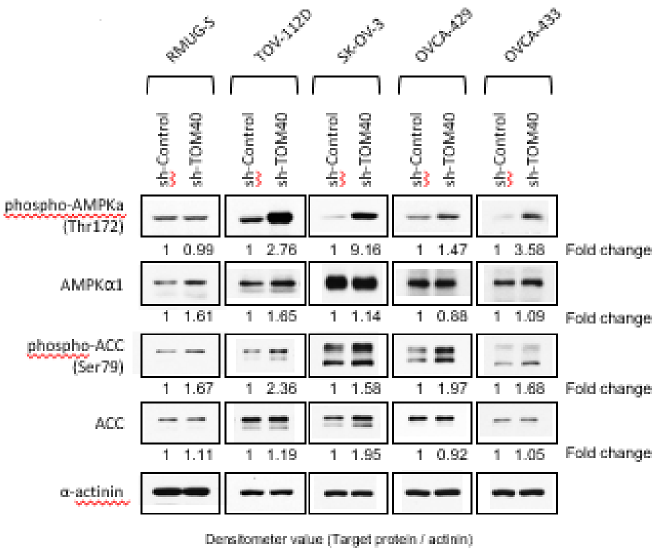

Blot no.

1	6	11	16	21
2	7	12	17	22
3	8	13	18	23
4	9	14	19	24
5	10	15	20	25

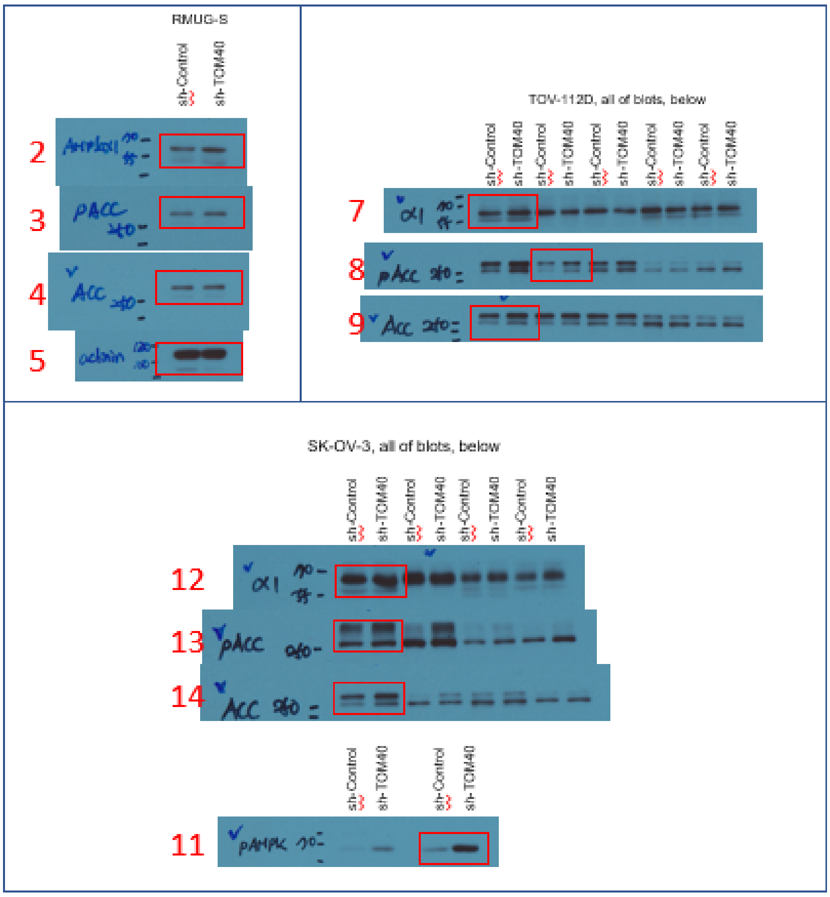

Figure S13. *Cont.*

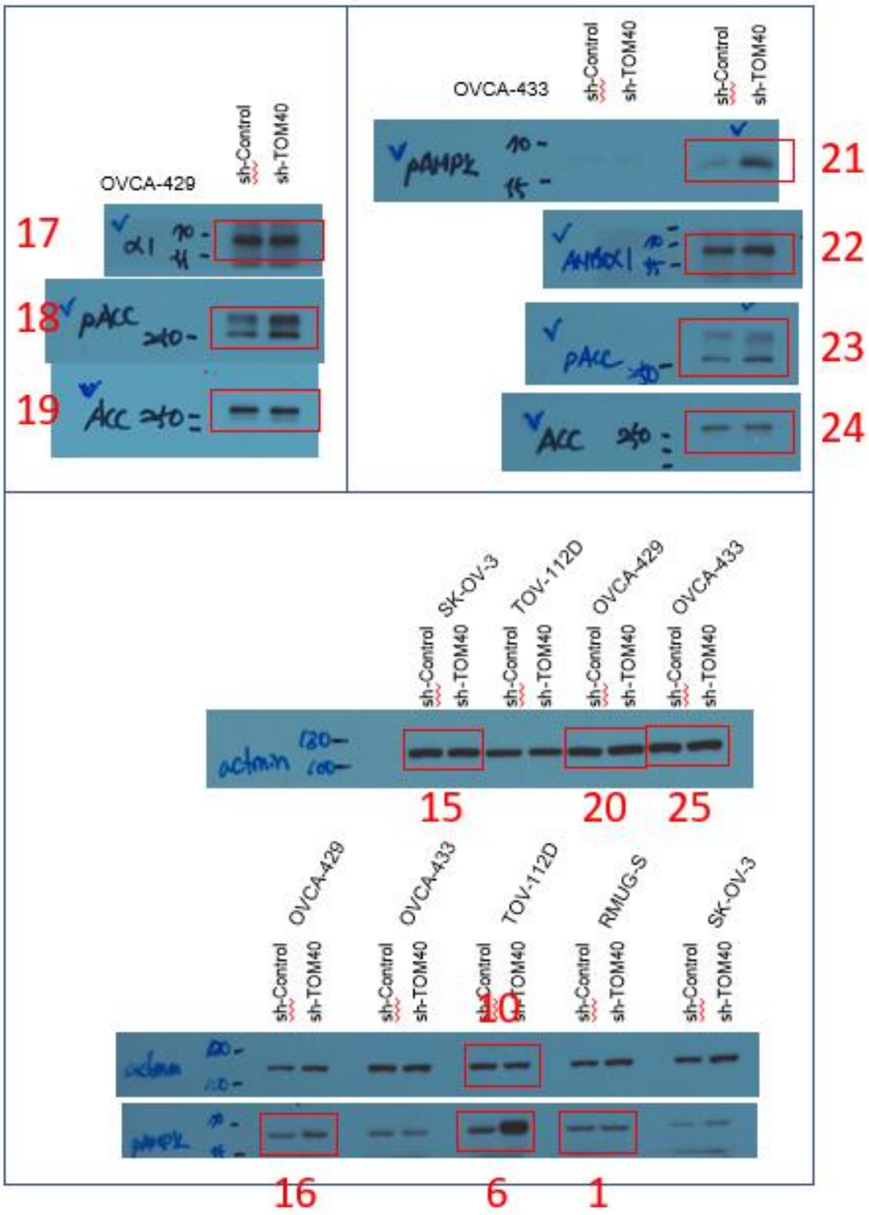

Figure S13. Original Western blots of figure 6A.

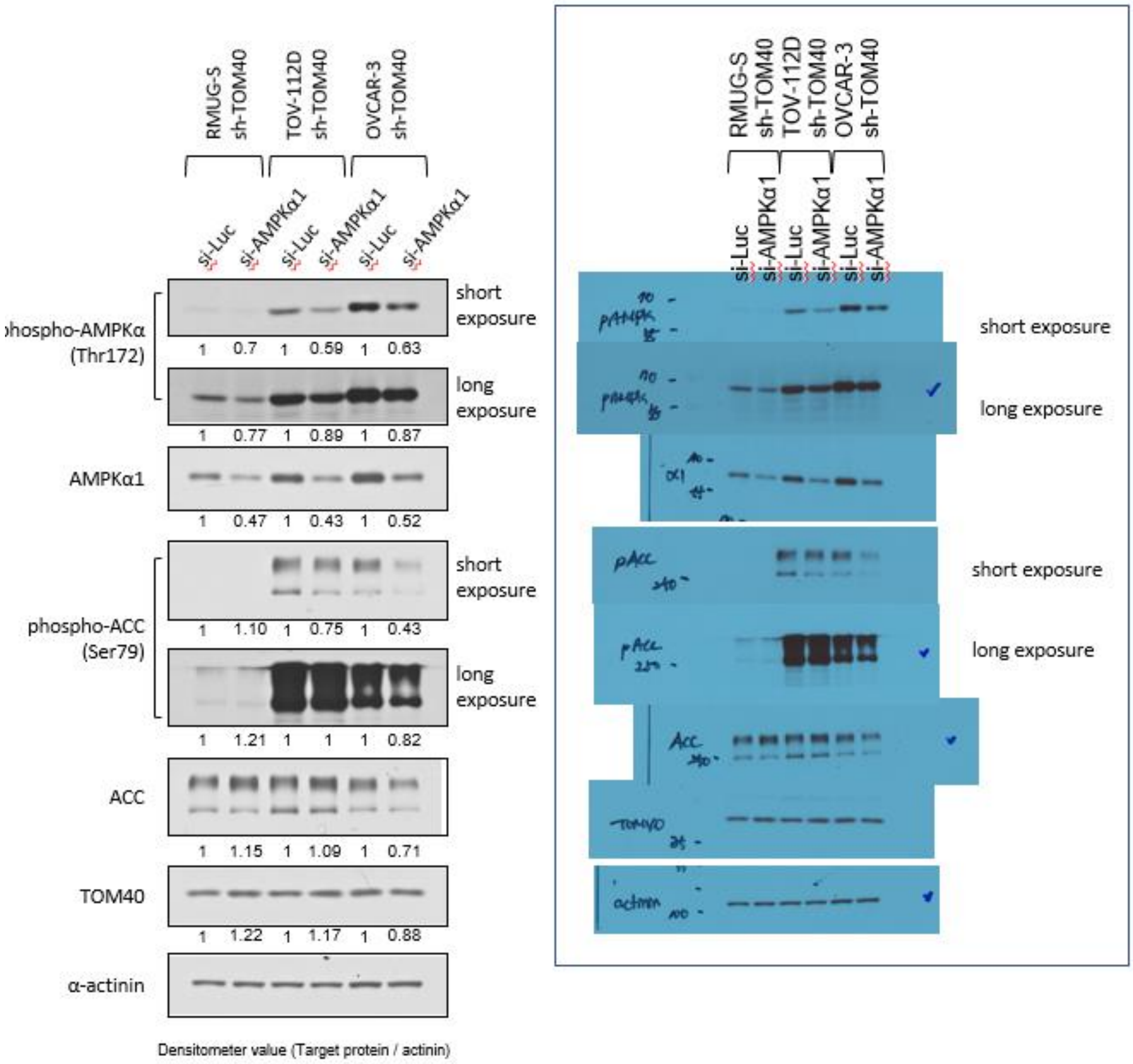

Figure S14. Original Western blots of figure 6B.

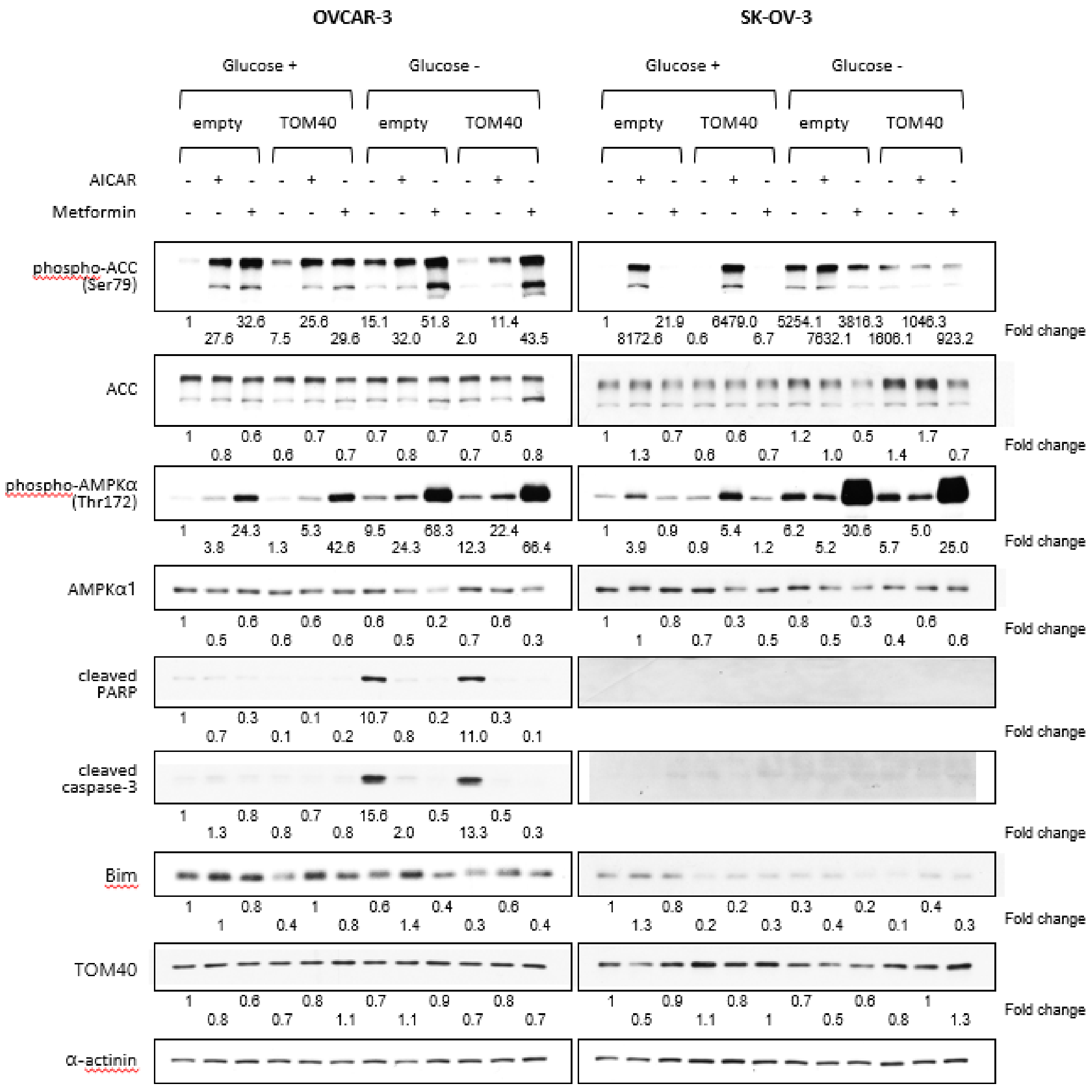

Densitometer value (Target protein / actinin)

Figure S15. *Cont.*

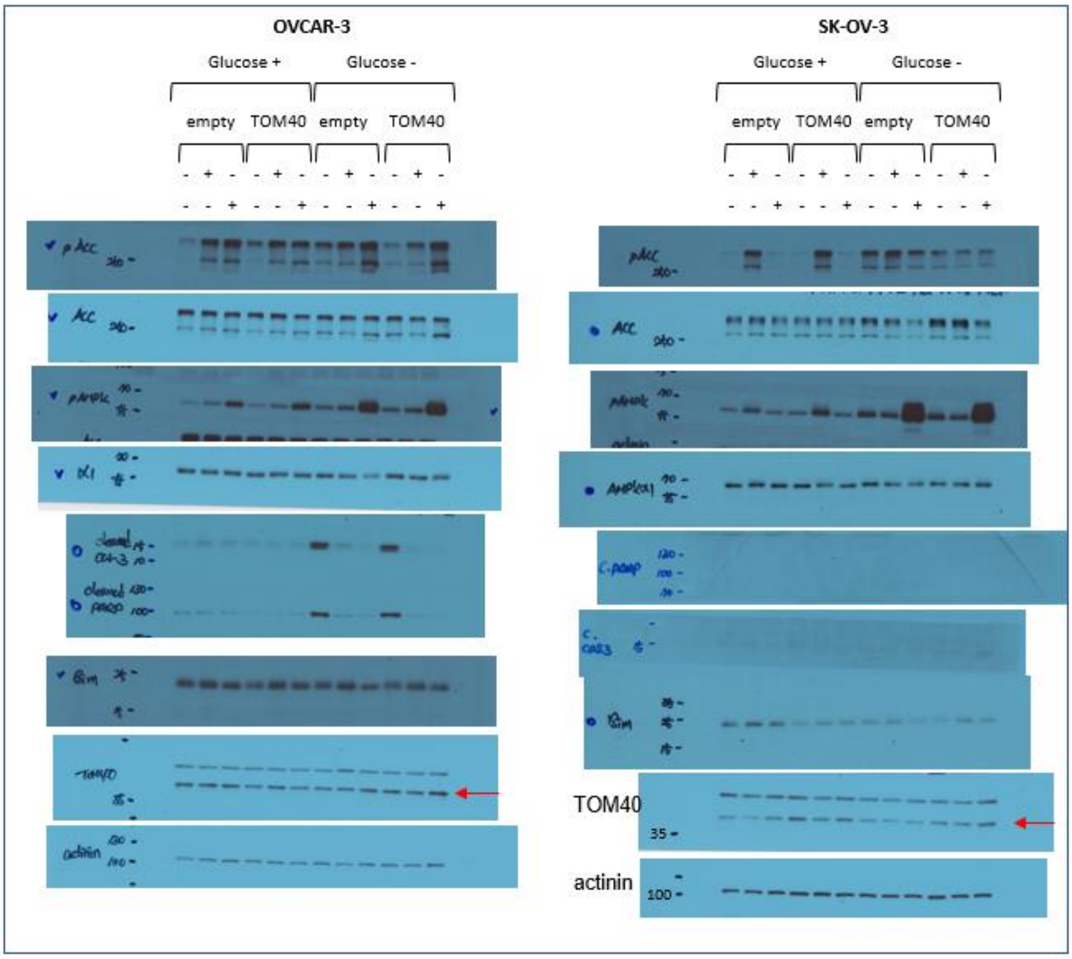

Figure S15. Original Western blots of figure 6E.

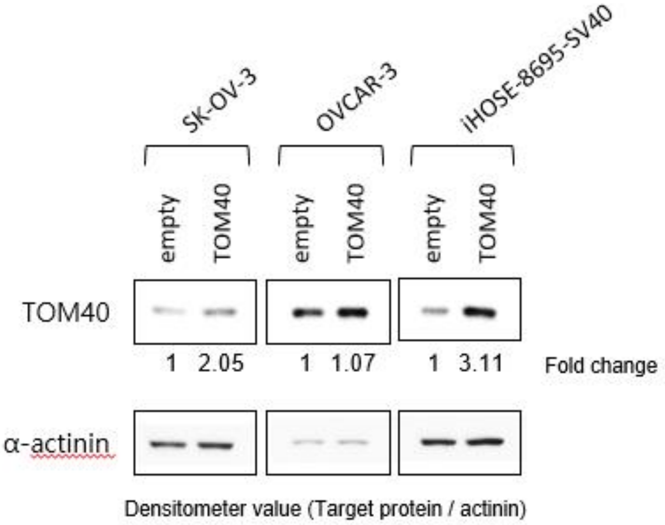

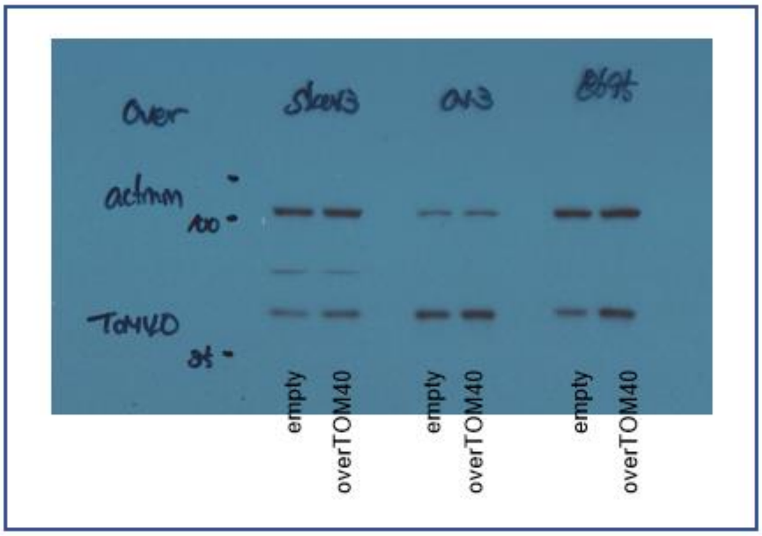

Figure S16. Original Western blots of figure S2.

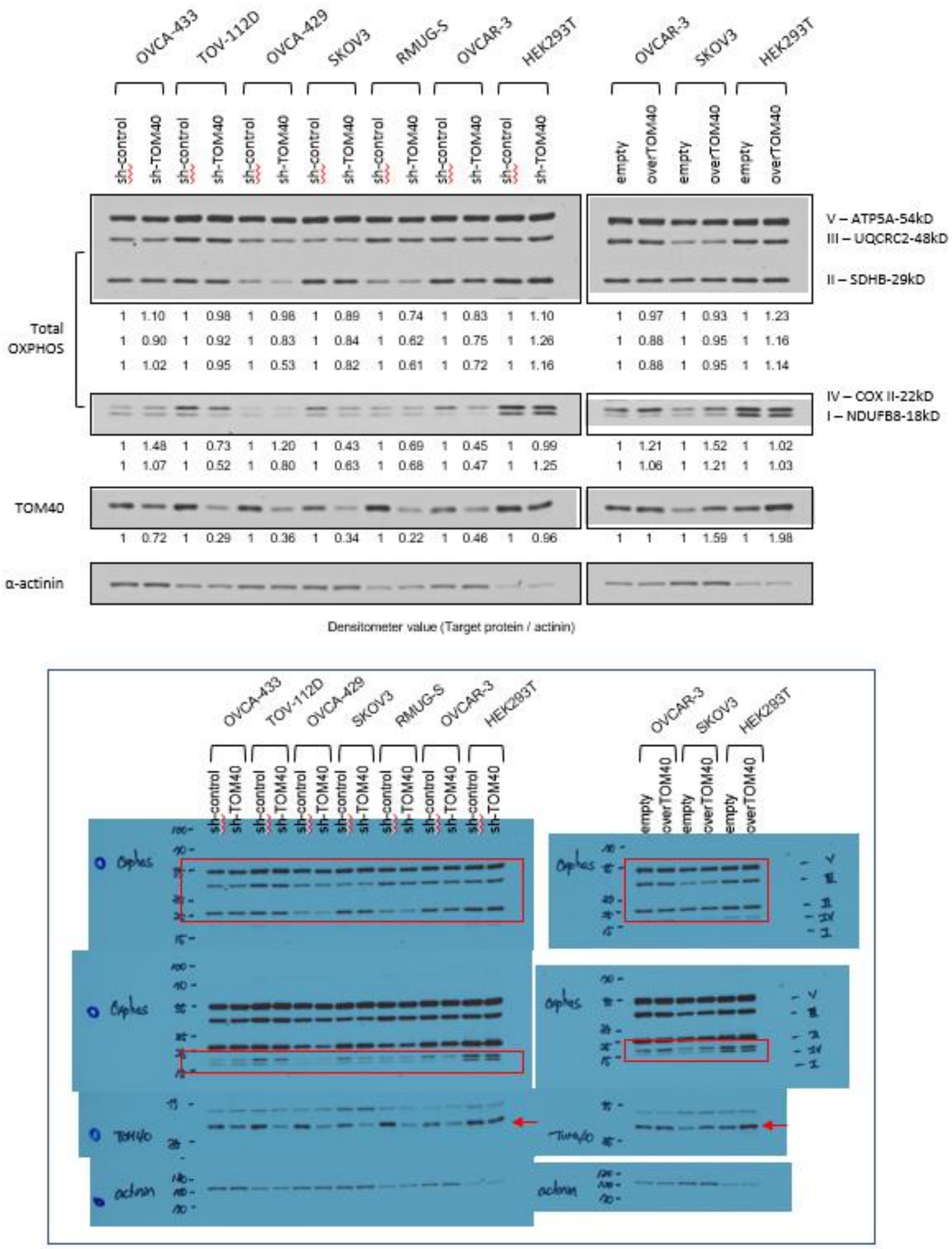

Figure S17. Original Western blots of figure S5.

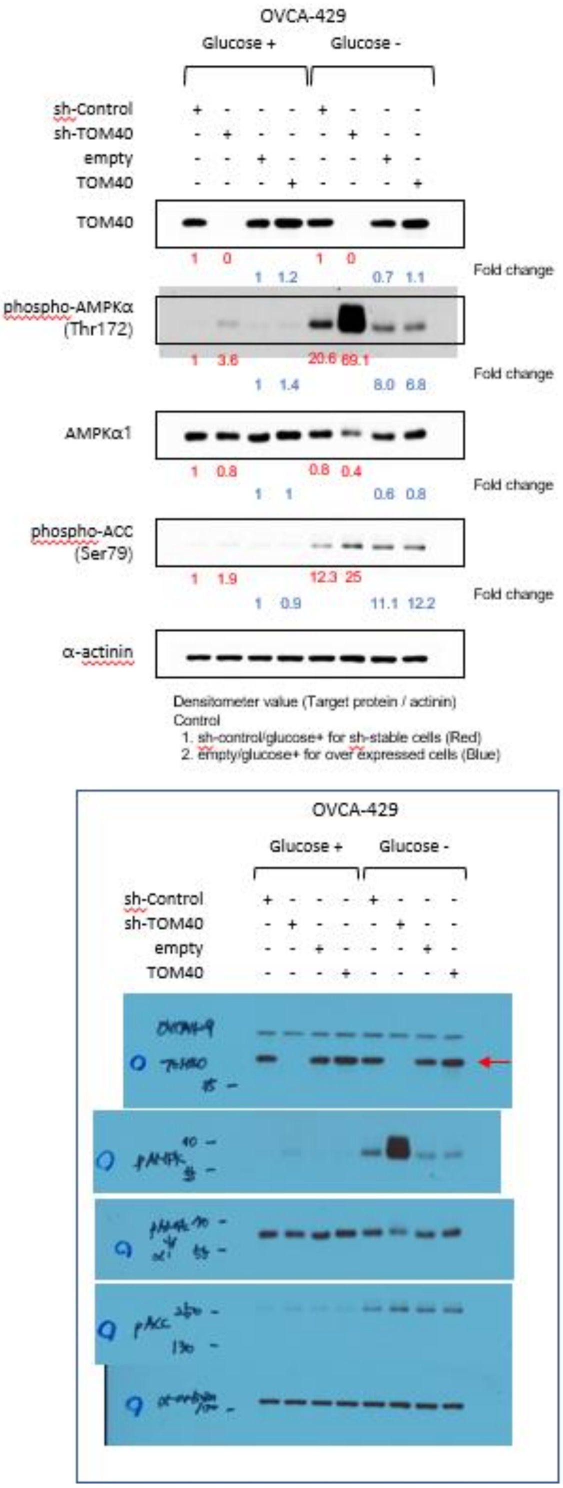

Figure S18. Original Western blots of figure S7.

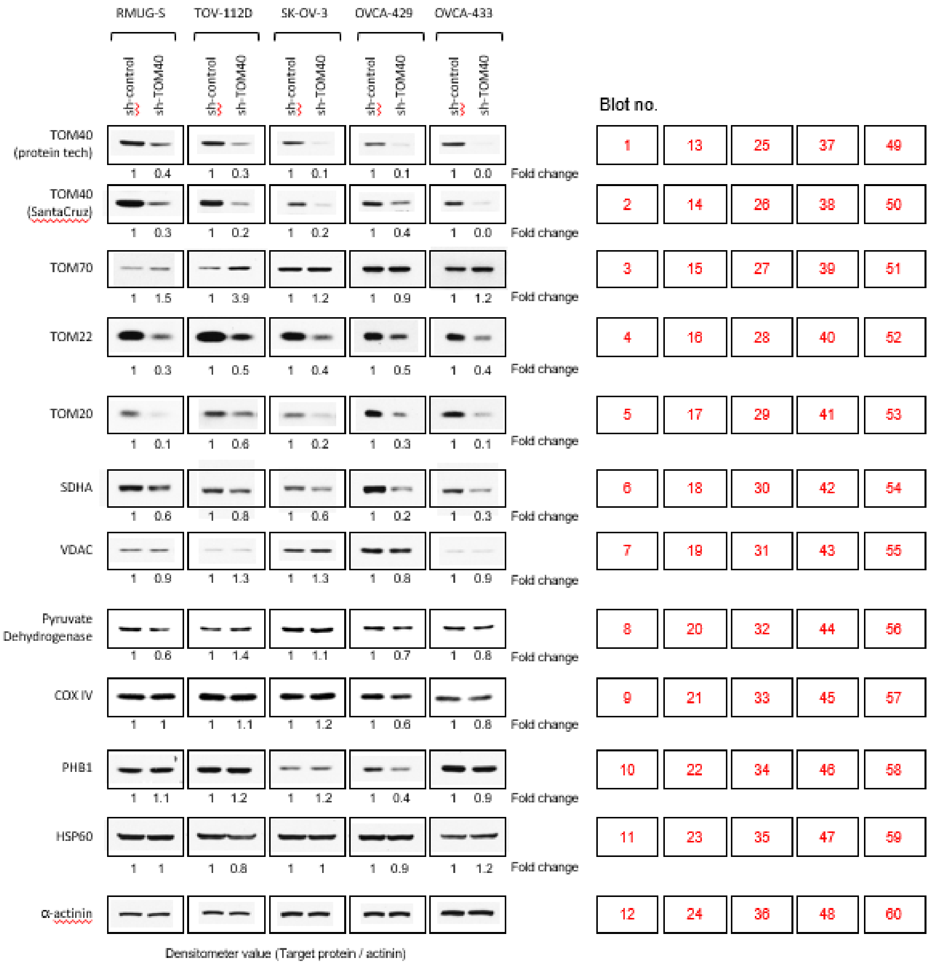

RMUG-S
TOV-112D
SK-OV-3
OVCA-429
OVCA-433
sh-control
sh-TOM40
Blot no.
TOM40 (protein tech)
1 0.4 1 0.3 1 0.1 1 0.1 1 0.0 Fold change
TOM40 (SantaCruz)
1 0.3 1 0.2 1 0.2 1 0.4 1 0.0 Fold change
TOM70
1 1.5 1 3.9 1 1.2 1 0.9 1 1.2 Fold change
TOM22
1 0.3 1 0.5 1 0.4 1 0.5 1 0.4 Fold change
TOM20
1 0.1 1 0.6 1 0.2 1 0.3 1 0.1 Fold change
SDHA
1 0.6 1 0.8 1 0.6 1 0.2 1 0.3 Fold change
VDAC
1 0.9 1 1.3 1 1.3 1 0.8 1 0.9 Fold change
Pyruvate Dehydrogenase
1 0.6 1 1.4 1 1.1 1 0.7 1 0.8 Fold change
COX IV
1 1 1 1.1 1 1.2 1 0.6 1 0.8 Fold change
PHB1
1 1.1 1 1.2 1 1.2 1 0.4 1 0.9 Fold change
HSP60
1 1 1 0.8 1 1 1 0.9 1 1.2 Fold change
α-actinin
Densitometer value (Target protein / actinin)
1 13 25 37 49
2 14 26 38 50
3 15 27 39 51
4 16 28 40 52
5 17 29 41 53
6 18 30 42 54
7 19 31 43 55
8 20 32 44 56
9 21 33 45 57
10 22 34 46 58
11 23 35 47 59
12 24 36 48 60

Figure S19. *Cont.*

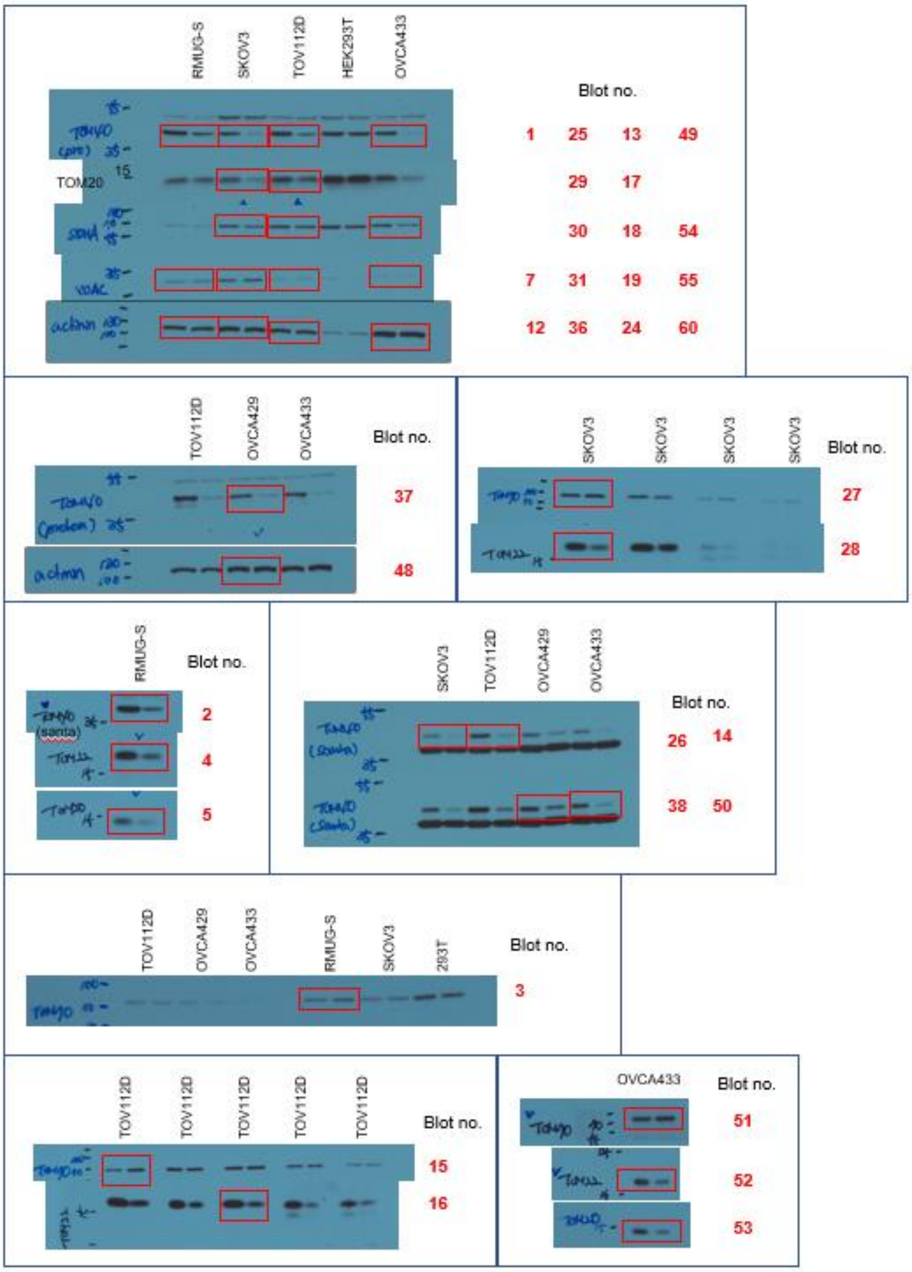

Figure S19. *Cont.*

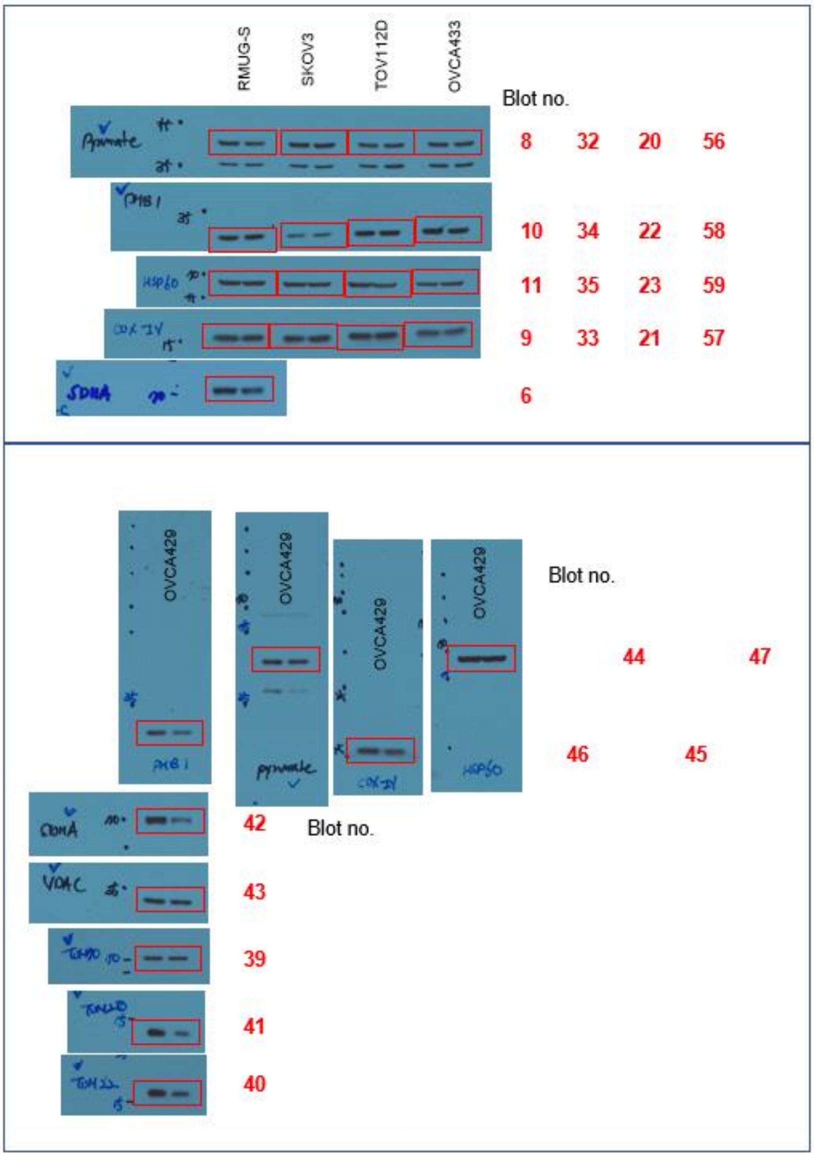

Figure S19. Original Western blots of figure S8A.

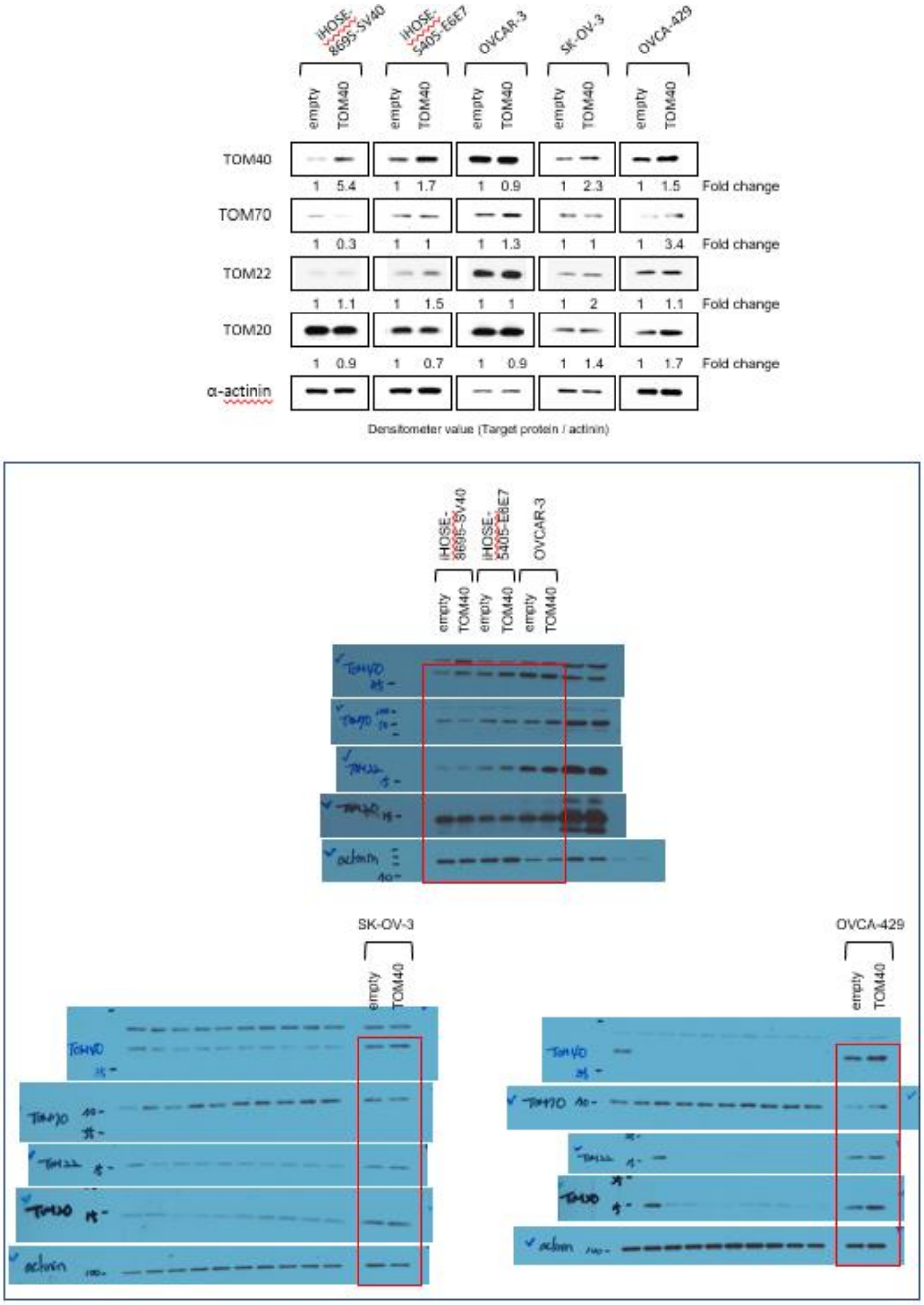

Figure S20. Original Western blots of figure S8C.

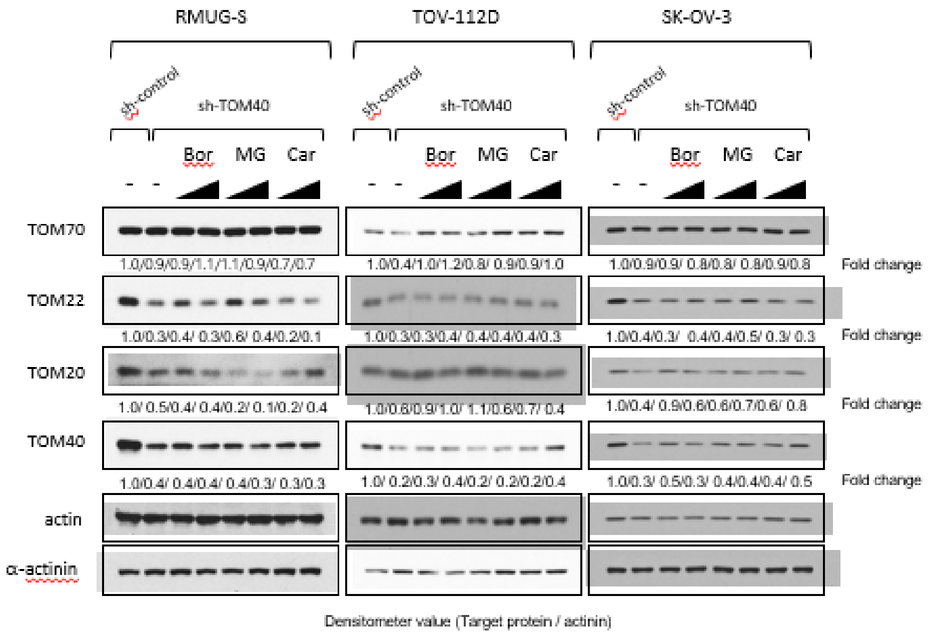
RMUG-S
TOV-112D
SK-OV-3
sh-control
sh-TOM40
Bor
MG
Car
TOM70
1.0/0.9/0.9/1.1/1.1/0.9/0.7/0.7
1.0/0.4/1.0/1.2/0.8/ 0.9/0.9/1.0
1.0/0.9/0.9/ 0.8/0.8/ 0.8/0.9/0.8
Fold change
TOM22
1.0/0.3/0.4/ 0.3/0.6/ 0.4/0.2/0.1
1.0/0.3/0.3/0.4/ 0.4/0.4/0.4/0.3
1.0/0.4/0.3/ 0.4/0.4/0.5/ 0.3/ 0.3
Fold change
TOM20
1.0/ 0.5/0.4/ 0.4/0.2/ 0.1/0.2/ 0.4
1.0/0.6/0.9/1.0/ 1.1/0.6/0.7/ 0.4
1.0/0.4/ 0.9/0.6/0.6/0.7/0.6/ 0.8
Fold change
TOM40
1.0/0.4/ 0.4/0.4/ 0.4/0.3/ 0.3/0.3
1.0/ 0.2/0.3/ 0.4/0.2/ 0.2/0.2/0.4
1.0/0.3/ 0.5/0.3/ 0.4/0.4/0.4/ 0.5
Fold change
actin
α-actinin
Densitometer value (Target protein / actinin)

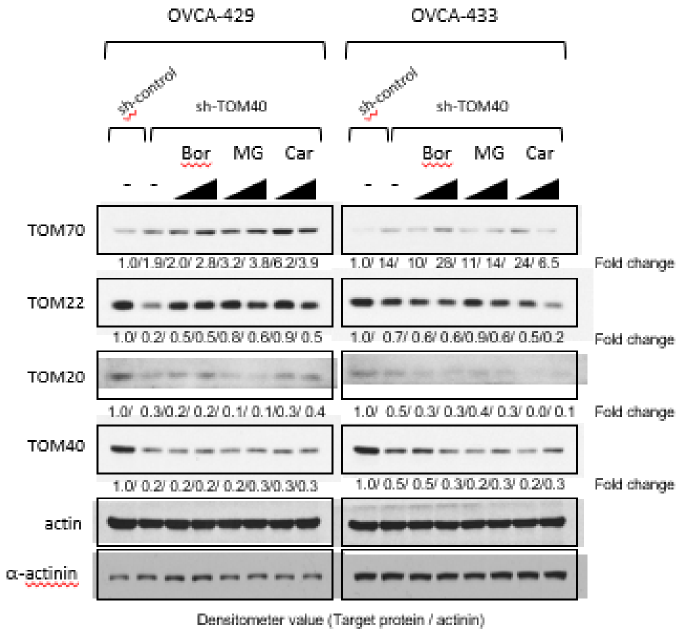
OVCA-429
OVCA-433
sh-control
sh-TOM40
Bor
MG
Car
TOM70
1.0/1.9/2.0/ 2.8/3.2/ 3.8/6.2/3.9
1.0/ 14/ 10/ 28/ 11/ 14/ 24/ 6.5
Fold change
TOM22
1.0/ 0.2/ 0.5/0.5/0.8/ 0.6/0.9/ 0.5
1.0/ 0.7/ 0.6/ 0.6/0.9/0.6/ 0.5/0.2
Fold change
TOM20
1.0/ 0.3/0.2/ 0.2/ 0.1/ 0.1/0.3/ 0.4
1.0/ 0.5/ 0.3/ 0.3/0.4/ 0.3/ 0.0/ 0.1
Fold change
TOM40
1.0/ 0.2/ 0.2/0.2/ 0.2/0.3/0.3/0.3
1.0/ 0.5/ 0.5/ 0.3/0.2/0.3/ 0.2/0.3
Fold change
actin
α-actinin
Densitometer value (Target protein / actinin)

Figure S21. *Cont.*

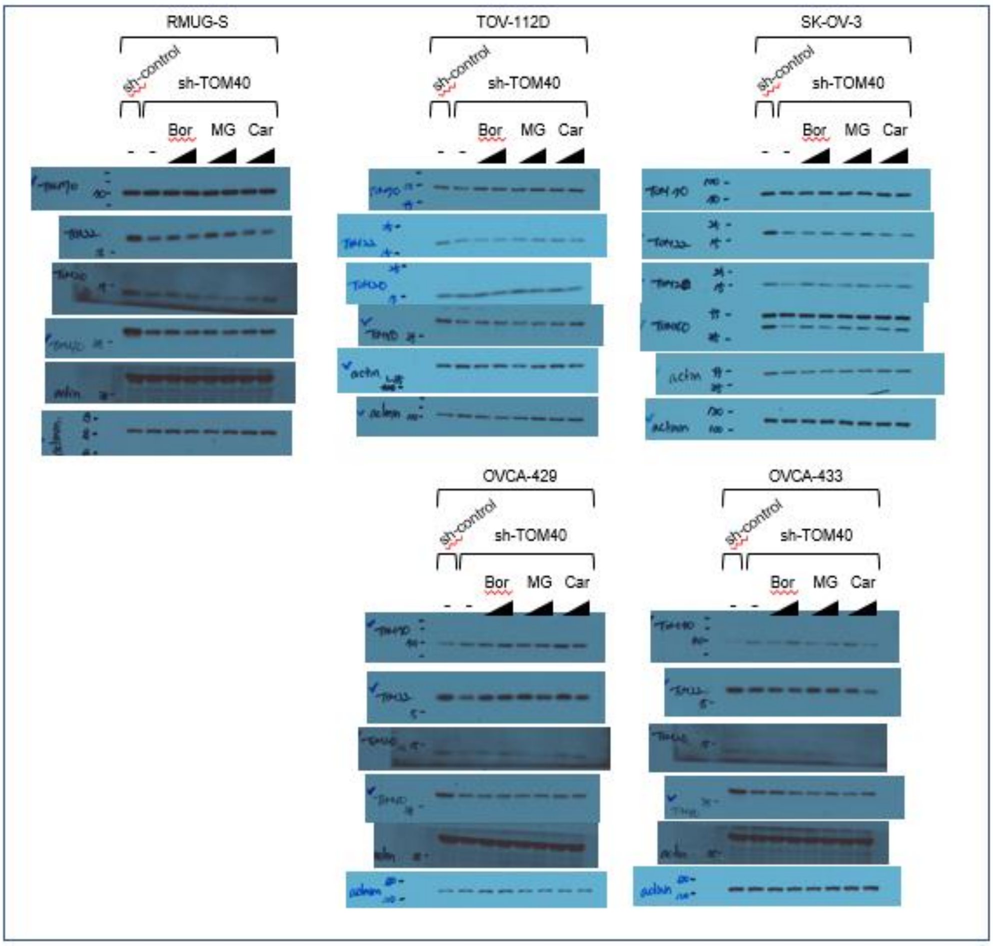

Figure S21. Original Western blots of figure S9A.

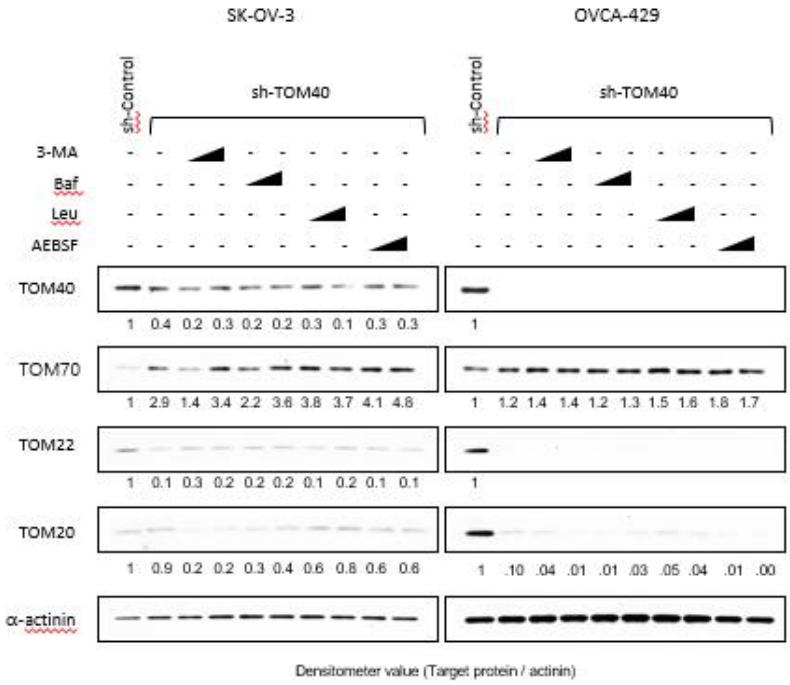

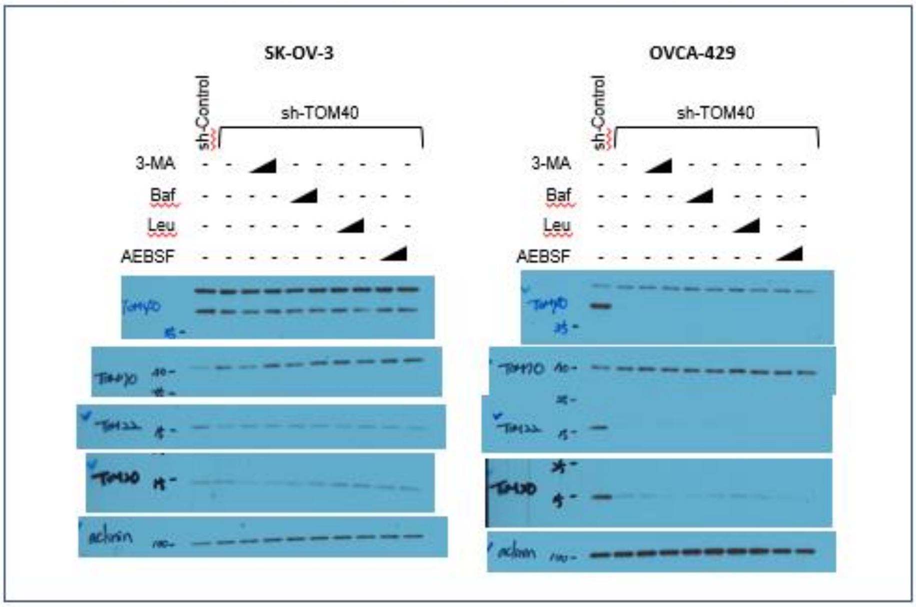

Figure S22. Original Western blots of figure S9B.

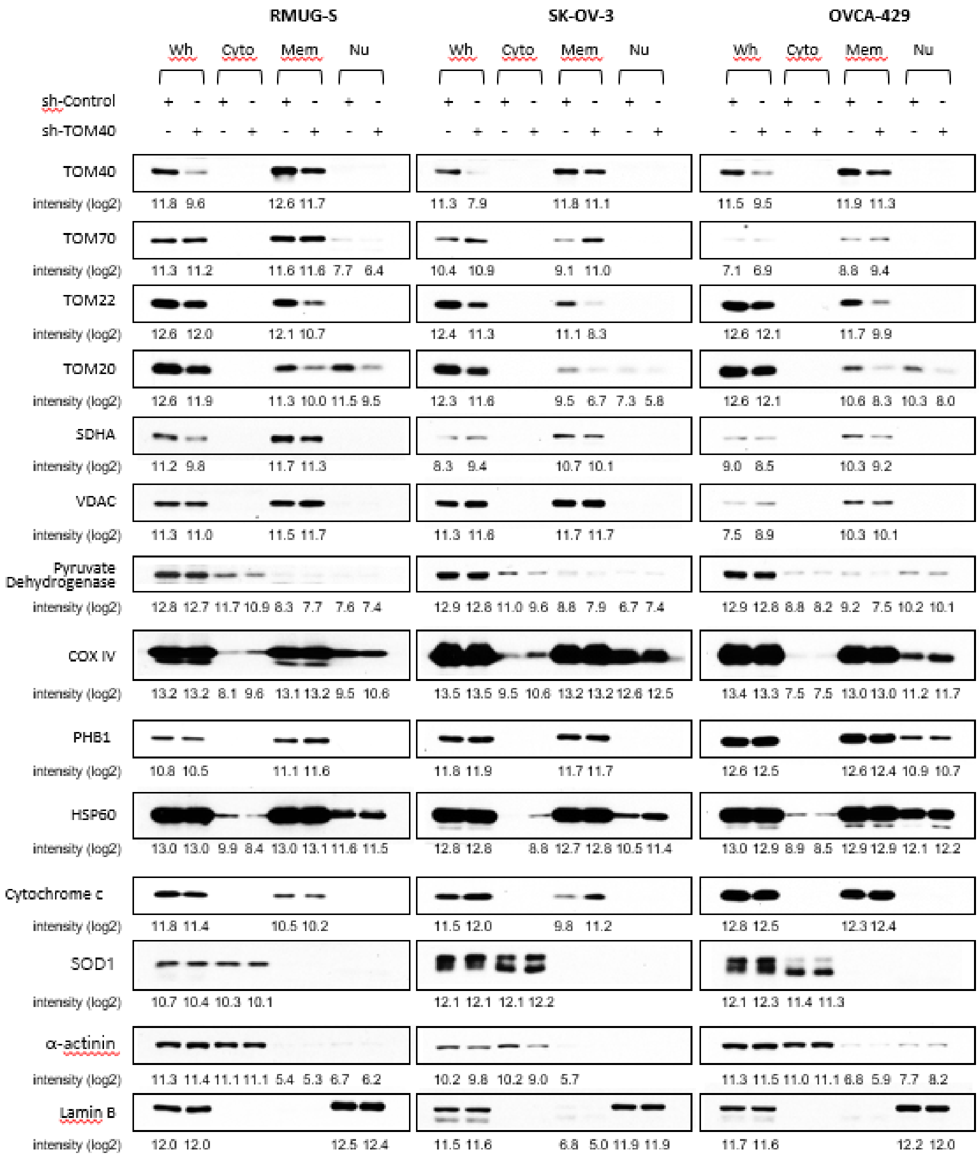

Densitometer value (log2)

Figure S23. *Cont.*

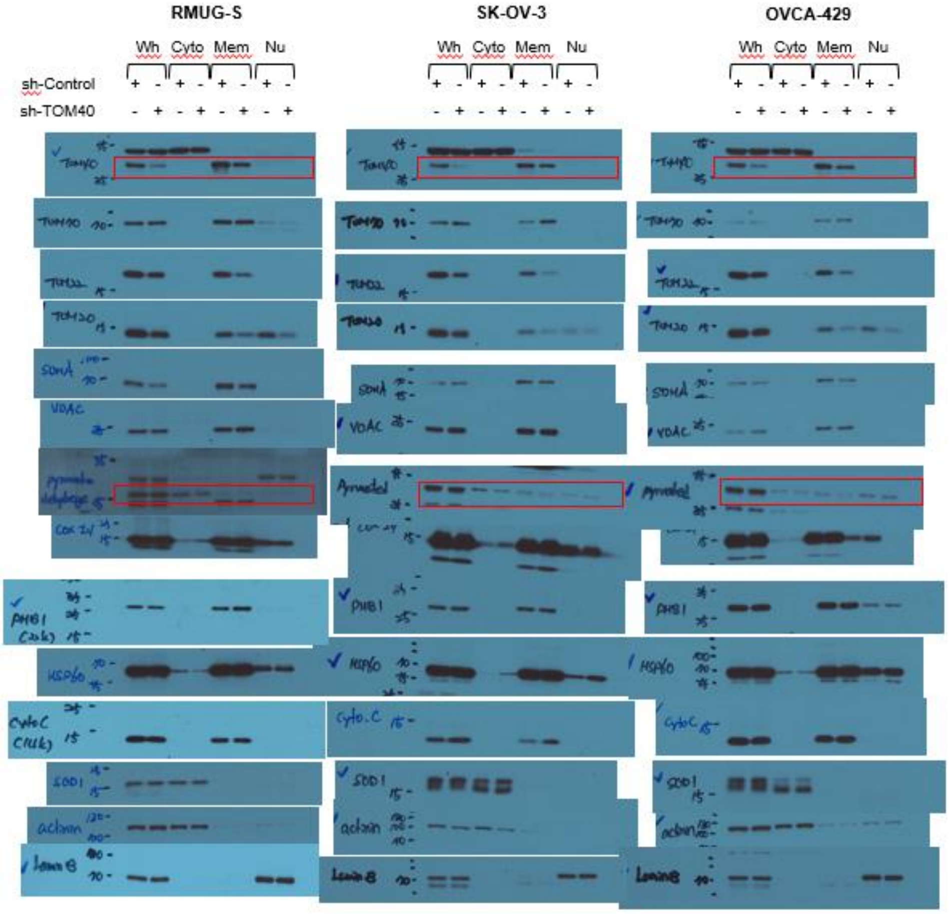

Figure S23. Original Western blots of figure S10.
